# The Medical Report of the Late L.G.B.

**Published:** 1920-03-06

**Authors:** 


### THE MEDICAL REPORT OF THE LATE L.G.B.

### The Last of a Famous Series.

The last of the reports of the Chief Medical Officer of the Local Government Board—a department now merged in the Ministry of Health—is signed by Sir George Newman, who took over the appointment early in 1919 and can therefore speak more freely on the achievements of his predecessors than one who had himself been responsible for the closing reports of the series, which stretched unbroken from 1871 to 1919. He opens with a well conceived and well written eulogy of Sir John Simon—not the lawyer, but the father of the new preventive medicine, who retired in 1876 and lived until 1904, when he died at the age of 87. He it was who initiated the reports, and, in fact, laid the foundations of the whole department so well and truly that his successors have been content to build on them without material alteration. He it was whose legacy to the nation, as Sir George Newman sees it, was "the gift of a method."

### The Duties of a Health Ministry.

The Central Medical Department, Simon said (and he advocated forty years ago the creation of a Ministry of Health), must always be endeavouring to learn. It must maintain and continually increase its own scientific efficiency as an advisory department. The knowledge upon which its advice is based shall always be the most exact and complete knowledge which at the time is possible. It must be incessantly watchful to recognise and appropriate for the public service all new experiences which concern the various most sanitary questions of the day, whether such experiences arise in its own administrative sphere or in the independent work of others. Where such experiences are fragmentary it shall integrate them by systematic special studies of its own. Lastly, in itself and in its publications it shall be foremost in a constantly advancing gain and proclamation of such new knowledge as may be of benefit to the public health. A high standard to aim at, indeed, one which may serve as an ideal for the new Ministry no less than for the Local Government Board. Nor were Simon's principles and methods whereby to carry out these conceptions any less wise or less lofty than the ideals themselves.

### The Great Influenza Pandemic.

The report deals in the main with the year 1918, though in certain respects it includes observations made down to the fusion in the Ministry of Health in June 1919. Naturally enough, it opens with a survey of the influenza epidemic of that year. This survey is mainly statistical and makes for that reason only dull reading: it appears that London suffered relatively less severely than New York, Chicago, San Francisco, and other leading American cities.

On malaria, trench fever and typhus, the rather pessimistic prognostications of the Board as to the results of demobilisation have not, happily, been borne out. How far this is due to the cautionary pronouncements issued is doubtful. A long report on cerebro-spinal fever (this also highly statistical) confirms the verdict of various researchers in recent years that infection with the organism of this disease is very much commoner than the clinical manifestations, which are so alarming and so deadly. In other words, only a minority of those who have the disease ever know it; but of that minority over sixty-five per cent. die.

Encephalitis lethargica also receives attention; 283 cases of this peculiar condition (provisionally regarded as a polio-encephalitis) were notified in the first quarter of 1919, and 287 cases in the second quarter. A clinical description of this obscure disease is given; and a footnote tells us that in November 1919 Dr. James McIntosh, at the London Hospital, succeeded in transmitting it to a monkey. Possibly, therefore, the natural history of the condition will shortly be much better understood than it has hitherto been. Meanwhile there is a tendency to diagnose this rare and obscure disease on much too flimsy a basis of evidence. Such errors the precise definitions here given should go far to correct.

A brief report on rabies comes next, followed by sections on plague and cholera. The section on smallpox points out that, while owing to military service conditions the adult male population is better vaccinated than has been the case for some years, the female and child population are less protected than ever. There are helpful reports from various sources on the best methods of managing the arseno-benzol treatment of syphilis; considerable divergencies are disclosed between what different authorities consider the optimum routine plan, but a memorandum by a special Salvarsan Committee lays down sundry rules and cautions which may well be taken as sound guidance for those whose special experience of this treatment is not very extensive.

### The Past and the Future.

In a word, the last report of the Local Government Board series, if less epochal than some of its predecessors, shows a record of good work done and steady progress made; and in view of the strain of the last year of war, during which the health of the country was still adequately defended, the Board and its medical officer (then Sir George Newsholme) are entitled to great credit for their strenuous efforts. The whole of the staff, Sir George Newman included, have been transferred to the Ministry of Health; so that a continuance of the series of reports, based on an even wider conception of the public health, is assured for future years. It is to be hoped that the first report of the new series will be able to announce the non-fulfilment of the forecasts of another influenza scourge.

